# *Helicobacter pylori* and unignorable extragastric diseases: Mechanism and implications

**DOI:** 10.3389/fmicb.2022.972777

**Published:** 2022-08-04

**Authors:** Junjian He, Yunyi Liu, Qin Ouyang, Rongxing Li, Jie Li, Weiyan Chen, Weichao Hu, Lijiao He, Qiyu Bao, Ping Li, Changjiang Hu

**Affiliations:** ^1^Department of Gastroenterology, Xinqiao Hospital, Army Medical University, Chongqing, China; ^2^Department of Medicinal Chemistry, College of Pharmacy, Army Medical University, Chongqing, China; ^3^Department of Foreign Languages, Army Medical University, Chongqing, China; ^4^Institute of Cardiovascular Diseases, Xinqiao Hospital, Army Medical University, Chongqing, China

**Keywords:** *Helicobacter pylori*, extragastric diseases, pathological mechanism, systemic inflammation, molecular mimicry

## Abstract

Considered as the most popular pathogen worldwide, *Helicobacter pylori* is intensively associated with diverse gastric diseases, including gastric ulcers, chronic progressive gastritis, and gastric cancer. Aside from its pathogenic effect on gastric diseases, growing evidences reveal that *H. pylori* may be related to numerous extragastric diseases. In this article, we reviewed recent studies and systematically elucidated that *H. pylori* may interfere with many biological processes outside the stomach and influence the occurrence of various extragastric diseases. Many epidemiological studies have indicated that *H. pylori* plays a pathogenic role in COVID-19, atherosclerosis, hyperemesis gravidarum and several other extragastric diseases, while the effect of *H. pylori* is currently under investigation in gastroesophageal reflux disease, asthma, and inflammatory bowel disease. Moreover, we also summarized the possible pathogenic mechanisms of *H. pylori* that may be related to chronic systemic inflammation and molecular mimicker. Taken together, this review provides a new perspective on the role of *H. pylori* in extragastric diseases and explores the possible mechanisms, which may help guide clinical treatment.

## Introduction

*Helicobacter pylori* is recognized as the most popular human pathogen, which infects nearly half of the population worldwide (approximately 4.4 billion people) (Hooi et al., 2017). Exposure to *H. pylori* may bring about lifelong chronic progressive gastritis, and 1–10% of infected individuals will have clinical complications, including gastric intestinal metaplasia, peptic ulcer disease, atrophy of gastric mucosa, gastric cancer (GC), and mucosa-associated lymphoid tissue (MALT) lymphoma (Yamaoka, 2018). The World Health Organization (WHO) has categorized *H. pylori* as one of the Class 1 carcinogens (No authors listed, 1994; Plummer et al., 2015). Previous studies have mostly focused on the role of *H. pylori* in inflammation and tumor development of the stomach. Several clinical trials have proven that the eradication of *H. pylori* reduces the incidence of GC (Lee et al., 2016) and atrophic gastritis (Choi et al., 2018). Management of epithelial precancerous conditions and lesions in the stomach (MAPS II) guideline in 2019 (Pimentel-Nunes et al., 2019) recommended prevention aims for *H. pylori* due to its role in gastric carcinogenesis, precancerous and early cancer lesions. Almost all previous clinical studies on *H. pylori* have suggested *H. pylori* eradication for patients suffering from gastric and intestinal metaplasia or chronic atrophic gastritis.

However, growing evidences reveal that *H. pylori* infection may be related to numerous extragastric diseases of various systems throughout the human body in addition to the pathogenetic effects on gastric diseases. For example, *H. pylori* has been described to be related to some blood system diseases. A separate meta-analysis of 15 observational studies proved that iron deficiency anemia (IDA) was more common among *H. pylori*-positive individuals than *H. pylori*-negative controls (OR = 2.2; 95% CI = 1.5–3.2) (Qu et al., 2010). *H. pylori* infection was also found to be more prevalent in adolescents suffering from IDA (Xia et al., 2012). In the reproductive system, a more significant incidence of *H. pylori* in pregnant women suffering from hyperemesis gravidarum was observed in a meta-analysis (Li et al., 2015). Some endocrine and metabolic diseases are also closely related to *H. pylori*. As shown in a meta-analysis, *H. pylori*-positive subjects with type 1 diabetes had a higher level of glycosylated hemoglobin than uninfected patients (Dai et al., 2015). Apart from the diseases mentioned above, *H. pylori* infection may also cause disorders in many other human systems (Razuka-Ebela et al., 2018). Moreover, studies on pathogenic mechanisms have shown that *H. pylori* can stimulate macrophages, T cells, B cells and other inflammatory cells to accelerate chronic systemic inflammation, interfere with normal physiological processes and ultimately becomes a crucial risk factor for atherosclerosis, insulin resistance, etc. (Franceschi et al., 2014). Similar antigens between *H. pylori* and human tissues may also lead to vitamin B deficiency, pernicious anemia and atherosclerosis (Chmiela and Gonciarz, 2017). The latest American College of Gastroenterology (ACG) Clinical Guideline in 2017 proposed associations between numerous extragastric disorders and *H. pylori* infection, aiming at raising the concern amid clinical workers to attach great importance on *H. pylori* and confronting these diseases in clinical practice (Chey et al., 2017).

In this article, we aim to elucidate the correlation of *H. pylori* and many extragastric diseases, which is necessary to refine the understanding of the pathogenic processes of *H. pylori* and help improve clinical prognosis and guide management. We reviewed latest studies and found that *H. pylori* may be associated with several extragastric diseases of various systems throughout the human body. In addition, we also explored the promising pathogenic mechanisms of *H. pylori* infection. Ultimately, we sought to improve and refine clinical guidelines and benefit patients suffering from the mentioned extragastric diseases and *H. pylori* infection.

## Respiratory disease

The relation of *H. pylori* infection with asthma has attracted extensive attention. For example, Zuo et al. (2021) found that *H. pylori* had a protective effect on allergic asthma by regulating Thl7/Tregs and the Th1/Th2 balance, reducing HSP70 and DCs, stimulating TLRs, and inhibiting gastroesophageal reflux. There are three well-known hypotheses related to the pathogenesis, including the gut-lung axis theory, the “disappearing microbiota” hypothesis and the hygiene hypothesis, all of them supporting the protective effect of *H. pylori*. In addition, therapeutic products made by *H. pylori* (such as *H. pylori* extract) have also been utilized to treat and prevent asthma. Perinatal *H. pylori* exposure reduced inflammation of the allergic airway in the offspring as well, providing a promising target for interventional therapy of asthma (Zuo et al., 2021). *H. pylori* can modulate anti-Th2 inflammation activity through neutrophil-activating protein (NAP) and contribute to allergic asthma, and purifying rNAP before sensitization can significantly reduce the accumulation of eosinophils in the lung tissue of asthmatic mice. It is worth noting that *H. pylori* treatment decreases the levels of IL-4, IL-13, and serological IgE, and increases the levels of IL-10 and IFN-γ (Zhou et al., 2017). This study suggests that eradication of *H. pylori* may have a preventive effect on the suppression of allergic asthma. However, it was not supported that *H. pylori* or its specific antigens provided protective antigens that reduced the occurrence of allergic asthma in a meta-analysis (Miftahussurur et al., 2017). Similarly, another cohort study published in 2017 showed that *H. pylori* was significantly associated with a 1.38-fold increased risk of asthma in adults. In addition, the risk of asthma in adults with *H. pylori* infection was still 1.85 times higher than that in *H. pylori* uninfected people (Wang et al., 2017). Thus, the protective effect of *H. pylori* on allergic asthma is controversial.

*Helicobacter pylori* may also promote the progression and evolution of chronic obstructive pulmonary disease (COPD). *H. pylori*-positive subjects showed a lower FEV1 (L) at baseline than *H. pylori*-negative patients, although no significant discrepancy in the decline rate between the two groups (*p*-value = 0.35) was shown (Sze et al., 2015). Socioeconomic status (SES) is a prognostic indicator for COPD. Interestingly, this study also found that years of education (on behalf of SES during childhood) were intensively associated with *H. pylori* status and might have effects on adult height. However, no significant difference was found in *H. pylori* seropositivity between individuals with GOLD 1 (global initiative for chronic obstructive lung disease) and GOLD 2 severity (Sze et al., 2015). A cohort study involving 3,619 subjects showed that neither *H. pylori* infection nor eradication treatment was related to COPD progression or lung dysfunction on a general population health screen. In summary, *H. pylori* may not be an intensively aggravated factor in lung function or COPD (Lee et al., 2020).

It is worth noting that *H. pylori* infection may also be associated with COVID-19. A large number of emerging results show that people infected with *H. pylori* may be more vulnerable to severe form of COVID-19 (Balamtekin et al., 2021). Besides, the inflammatory activation caused by *H. pylori* infection may enhance the respiratory inflammatory response of COVID-19, recruit inflammatory cells and promote sustained production of TNF-α, IL-8, and IL-1β, as well as endothelial dysfunction markers such as V-CAM and ICAM, leading to subsequent virus-mediated acute lung injury. *H. pylori* may also aggravate acute respiratory distress syndrome (ARDS), which is a serious complication threatening numerous COVID-19 patients (Gonzalez et al., 2022). However, there was no significant difference in loss of smell, dyspnea, fever, and dry cough between COVID-19 patients with or without *H. pylori* infection. At present, there is no evidence showing that *H. pylori* infection significantly increases the risk of chronic pulmonary fibrosis and COPD among patients with COVID-19 (Balamtekin et al., 2021). The possible reason may be that *H. pylori* infection only affects the acute progression of COVID-19, but not the chronic course.

Studies have found that *H. pylori* pathogen-derived proteins (such as VacA) are found in lung biopsy specimens and bronchoalveolar lavage fluid of lung cancer. These proteins can aggravate the progress of airway diseases, promote the *H. pylori* infection inflammatory status (anti-*H. pylori* IgG and IgM) and recruit B cells, and finally accelerate the occurrence of lung cancer. Besides, eradication of *H. pylori* was significantly correlated with the decrease of lung cancer marker CEA. This explained that *H. pylori* may be of benefit for the treatment of lung cancer (Xu et al., 2018a). Of concern, there is a currently ongoing clinical trial investigating the association between *H. pylori* strain specific blood biomarkers and lung cancer risk (PLCO2019-1026), which may help understand of *H. pylori* infection and lung cancer risk, identify markers for lung cancer risk, and provide new information for a feasible cancer prevention strategy.

Although recent studies suggested an association between *H. pylori* infection and respiratory diseases, further studies are necessary to confirm a causal relationship. Moreover, the roles of other risk factors, such as air pollution or smoking habits, as well as the latent molecular mechanisms should also be considered (GonzAlez et al., 2018; Supplementary Table S1).

## Heart and circulatory disease

The association of *H. pylori* infection with coronary artery disease has also been investigated. One study showed that *H. pylori* infection significantly reduced endothelium-dependent flow-mediated vasodilation in a young group and strongly repressed acetylcholine-induced endothelium-dependent aortic relaxation without altering nitroglycerin-induced endothelium-dependent vascular relaxation in mice. In addition, *H. pylori* eradication in both human subjects and mice obviously improved endothelium-dependent vasodilation (Xia et al., 2020). Infection with serum CagA+ *H. pylori* can induce cardiovascular disease and coronary heart disease (Sharma and Aggarwal, 2015). Mechanisms by which CagA+ *H. pylori* causes atherosclerosis include increasing the production of COX-1/2 from the vascular endothelium, thereby stimulating the synthesis of thromboxane A2 (TXA2) and prostaglandin to induce platelet aggregation. In addition, *H. pylori* releases many cytokines, including interleukin-6 (IL-6), tumor necrosis factor-α (TNF-α), IL-1 and free radicals, causing atherosclerosis and oxidative stress. Furthermore, an aberrant immune reaction is considered to play a role in atherosclerotic plaque rupture and destabilization by the cross-reactivity between antibodies and CagA vascular wall antigens (de Boer et al., 2000; Byrne et al., 2003; Guo et al., 2007; Feletou et al., 2011). Therefore, as *H. pylori* infection can lead to endothelial dysfunction, dyslipidemia and hyperhomocysteinemia, *H. pylori* eradication therapy is recommended as a possible secondary cardiovascular prevention strategy (Zuin et al., 2016).

Myocardial infarction (MI) is the most dire and serious outcome for patients with CAD due to its fatal influence on survival quality. A meta-analysis including more than 20,000 subjects and 26 studies found that *H. pylori* infection is a risk factor for MI, even among young participants (Liu et al., 2015).

A cohort study that included 12,836 participants showed that *H. pylori* may also significantly increase the risk of carotid atherosclerosis in Chinese men under 50 years old (Zhang et al., 2019). Another study indicated that non-alcoholic fatty liver disease (NAFLD) caused by infection with *H. pylori* increases the formation of carotid artery plaques (Yu L. Y. et al., 2019).

After adjusting for potential cofactors, a trial that included 5,168 study participants revealed an association between high blood pressure and *H. pylori*. In this study, *H. pylori* was related to an increased risk of hypertension (95% CI = 1.04–1.46; OR = 1.23). Compared with individuals without *H. pylori* infection, infected subjects showed a 0.735 mmHg increase in diastolic blood pressure (95% CI = 0.101–1.369) and a 0.723 mmHg increase in mean arterial pressure (95% CI = 0.034–1.413) (Wan et al., 2018; Supplementary Table S1).

## Digestive disease

Eosinophilic esophagitis (EoE) is a kind of disease mediated by the immune response. A meta-analysis by Doulberis et al. (2020a) revealed that *H. pylori* infection is one of the protective factors against EoE. However, in 2018, a prospective case–control study conducted in 23 centers reported that *H. pylori* was not negatively associated with EoE, neither in adults nor in children (Molina-Infante et al., 2018). Thus, the effect of *H. pylori* infection on EoE still needs further study.

In developing countries, esophageal squamous cell carcinoma is a prevalent esophageal disorder. Currently, there is no definite evidence showing that *H. pylori* infection contributes to the incidence of esophageal squamous cell carcinoma. A meta-analysis of 35 studies with 345,886 participants indicated that there was no crucial association between esophageal squamous cell carcinoma and *H. pylori* infection (Gao et al., 2019). However, a study that included 95 esophageal squamous cell carcinoma patients showed a statistically significant negative association between esophageal squamous cell carcinoma and *H. pylori* infection via testing gastric biopsy materials from the patients (Poyrazoglu et al., 2017).

Some studies have proposed a different relationship between *H. pylori* and gastroesophageal reflux disease (GERD). An analysis of GERD patients found a higher prevalence of *H. pylori* infection among patients with peptic ulcers (Jie et al., 2019). In contrast, a prospective clinical study of 124 patients with GERD, revealed that *H. pylori* infection reduced esophageal acid exposure, enhanced lower esophageal sphincter pressure, and improved esophageal peristalsis. Thus, *H. pylori* may be protective factors for GERD (Liu et al., 2018). However, interestingly, *H. pylori* eradication did not increase the incidence of GERD. In summary, more studies are needed to determine this pathogenesis.

Several clinical trials have found a relationship between hepatocellular carcinoma (HCC) and *H. pylori*, which was detected in liver samples from individuals with HCC, but this presence cannot support a definite causal relationship (Okushin et al., 2018).

Cholelithiasis and chronic cholecystitis are quite prevalent worldwide. A meta-analysis found that the chronic cholecystitis/cholelithiasis group was more prevalent in *H. pylori* infected gallbladder than the control group in 17 studies (Wang et al., 2021).

The supposed role of *H. pylori* infection in gallstones and gallbladder polyps is still debated. A retrospective study showed that *H. pylori* infection was related to gallstones and gallbladder polyps in a Chinese population (Xu et al., 2018b), whereas this relation was not supported in another case–control matched study of a Chinese population (Zhang et al., 2020). Thus, the role of *H. pylori* in cholecystic polyps and gallstones requires further research.

Non-alcoholic fatty liver disease is a kind of liver injury that is induced by metabolic stress. A meta-analysis of 21 studies indicated that *H. pylori* infection was one of the factors contributing to NAFLD progression in the Asian population (Liu et al., 2019), but *H. pylori* infection was not an independent risk factor for NAFLD revealed by a cross-sectional study in China (Fan et al., 2018). One hypothesis is that *H. pylori* infection may cause chronic low-level systemic inflammation, which increases the concentration of inflammatory cytokines, such as IL-6 and TNF-α, stimulating IKK/NF-κB signaling and leading to insulin resistance. *H. pylori* infection may also restrain leptin release from white adipose tissue, which in turn leads to liver stearoyl-CoA desaturase, thereby stimulating fat and VLDL-C deposition in liver tissue. Another hypothesis is that *H. pylori* infection may cause dysbiosis of gastrointestinal flora, increase serum lipopolysaccharide, accelerate the systemic inflammatory response and increase the expression of IL-6, TNF-α, and C-reactive protein, which results in reduced lipoprotein activity followed by dyslipidemia (Cheng et al., 2017). Notably, an ongoing clinical study may contribute to reveal the risk of NAFLD due to *H. pylori* infection by investigating the genome-wide association of *H. pylori* infection (PLCO-989).

*Helicobacter pylori* infection might play a protective role in inflammatory bowel disease (IBD) reported by a meta-analysis (Imawana et al., 2020). Besides, another meta-analysis of clinical studies including 1,748 individuals, also indicated an association between CagA seropositivity and lower odds of IBD (Tepler et al., 2019; Supplementary Table S2).

Viral hepatis has also been found to be related to *H. pylori* infection. Esmat et al. (2012) found the existence of CagA gene of *H. pylori* in liver samples of patients with hepatitis C virus (HCV)-related chronic hepatitis. A multivariate analysis further indicated that positive anti-*H. pylori* antibody was independently and significantly related to cirrhosis in individuals with HCV-related chronic hepatitis (Queiroz et al., 2006). Moreover, clinical reports also suggested an association between *H. pylori* and HBV-related liver diseases. A meta-analysis of a Chinese population demonstrated that the infection rate of *H. pylori* in patients with HBV-related liver diseases had a positive relation with the increase of disease severity. In addition, the rate of *H. pylori* positivity in chronic HBV patients was 2.44-fold higher than that in healthy controls (Wang et al., 2016). Therefore, the prevalence of *H. pylori* may promote the progression of HBV-related liver diseases. However, the relationship between *H. pylori* infection and HAV is usually overestimated by confounding factors such as socio-economic status and age, and eliminating interference of these factors would reduce this correlation (BinSaeed, 2010).

**FIGURE 1:**
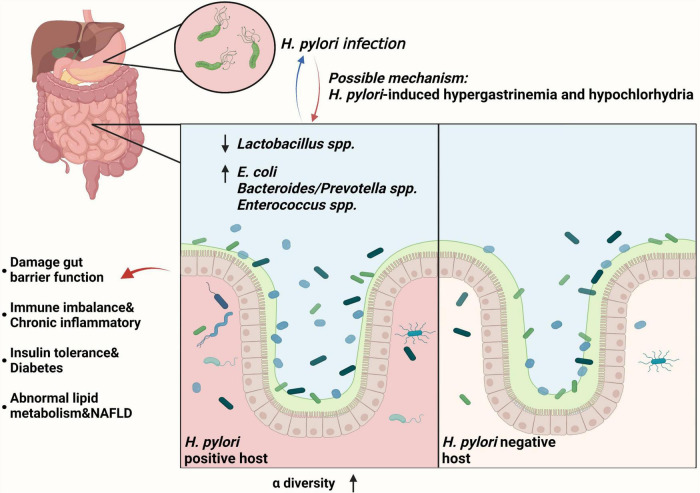
*Helicobacter pylori* infection can interact with the gut microbiome and affect extragastric diseases progression. With chronic *H. pylori* infection, gut microbiome showed a lower level of *Lactobacillus* spp. and a significant higher loads of *E. coli, Bacteroides/Prevotella* spp., and *Enterococcus* spp., increasing α-diversity of gut microbiota (Heimesaat et al., 2014). For the mechanism, gut microbiota changes may be triggered by *H. pylori*-induced gastric immune pathogenesis, including hypergastrinemia and hypochlorhydria (Lopetuso et al., 2014). Subsequently, low level of beneficial gut bacteria (such as *Lactobacillus* spp.) may lead to the proliferation of some harmful bacteria and damage gut barrier function (Sanders et al., 2019). This also leads to the immune imbalance and chronic inflammatory, insulin tolerance and diabetes, and abnormal lipid metabolism and NAFLD (He et al., 2016).

It has been found that *H. pylori* can interact with the gut microbiome and affect extragastric diseases progression. Heimesaat et al. (2014) found that with long-term *H. pylori* infection, gut microbiome showed a lower level of *Lactobacillus* spp. and a significant higher loads of *E. coli*, *Bacteroides/Prevotella* spp., and *Enterococcus* spp. than *H. pylori*-negative subjects. In addition, *H. pylori* permits more microorganisms to pass through the gastric acid barrier and colonize the distal gut, increasing gut microbiota diversity through hypergastrinemia and hypochlorhydria (Lopetuso et al., 2014). Subsequently, low level of beneficial gut bacteria (such as *Lactobacillus* spp.) may lead to the proliferation of some harmful bacteria and damage gut barrier function. This also causes the immune imbalance and mediates several chronic inflammatory diseases mentioned above (Sanders et al., 2019). Furthermore, *H. pylori* infection-related gut microbiome alternation may decrease insulin sensitivity and lead to diabetes, and may also lead to abnormal lipid metabolism, increasing the risk of NAFLD (He et al., 2016). Recovery of gut microbiome balance was observed after *H. pylori* eradication (Chen et al., 2021). Taken together, *H. pylori* can induce the gut microbiome alternation and lead to the progression of several extragastric diseases (Figure 1).

## Blood system disease

It is found that *H. pylori* infection is closely related to MALT lymphoma. *H. pylori*-induced T cells can promote macrophages to secrete APRIL, which is an important cytokine that promotes the progression of MALT lymphoma (Planelles et al., 2008; Zhang et al., 2015). *H. pylori* may also directly drive CagA protein into B cells, leading to increased Bcl-2 expression, activating extracellular signal-regulated kinase and inhibiting apoptosis, which finally promote MALT lymphoma progression (Kuo et al., 2014). Besides, CagA+ *H. pylori*-infected MALT lymphoma patients significantly delayed the progression of MALT lymphoma after *H. pylori* eradication treatment (Kuo et al., 2014). Infected by *H. pylori*, normal B cells are driven into malignant clones by three kinds of chromosomal translocations, t (14;18) (q32; q21), t (1;14) (p22; q32), and t (11;18) (q21; q21), activating NF-κB signaling and regulating apoptosis, inflammation, and immunity (Bertoni and Zucca, 2006; Ruskone-Fourmestraux et al., 2011; Bautista-Quach et al., 2012; Zullo et al., 2014). Among them, t (Dai et al., 2015; Miftahussurur et al., 2017) (q21; q21) may be conducive to the occurrence of MALT lymphoma (Streubel et al., 2006).

Many studies have proven that *H. pylori* infection leads to IDA. The Maastricht III European guidelines for people with unknown sarcopenic anemia recommend an *H. pylori* infection test and germ eradicate therapy (Malfertheiner et al., 2007). Flores et al. (2017) found that CagA protein is significant in alteration of iron metabolism in gastric adenocarcinoma cells of *H. pylori*-infected humans, and this is mediated by transferrin endocytosis and increasing iron uptake.

It has been reported that the lack of vitamin B12 absorption contributes to pernicious anemia and *H. pylori* also plays a role in this process. *H. pylori* infection changes intragastric pH, leading to vitamin B12 malabsorption (Cohen et al., 2000). In addition, *H. pylori* may also evoke an antigen similar to antibodies against the H+K+-adenosine triphosphate protein to inhibit vitamin B12 absorption (Claeys et al., 1998). Besides, an ongoing clinical study may help reveal the risk of vitamin B12 deficiency due to *H. pylori* infection by investigating the genome-wide association of *H. pylori* infection (PLCO-989).

The role of *H. pylori* in Idiopathic or Immune Thrombocytopenic Purpura (ITP) has also been investigated. A meta-analysis of six studies involving 241 patients proved that *H. pylori* eradication is an effective treatment for ITP patients (Kim B. J. et al., 2018). Lei et al. (2021) reported that *H. pylori* can promote platelet destruction in mice, and the mechanisms may be related to activating NF-κB/IL-17 signaling.

Antiphospholipid syndrome is characterized by both venous and arterial thrombosis, and often leads to abortions, premature birth, and preeclampsia. Cicconi et al. (2001) reported that after the eradication of *H. pylori*, the antiphospholipid syndrome of a case disappeared (Supplementary Table S3).

## Endocrine and metabolic disease

Diabetes is the most prevalent metabolic disorder worldwide, killing approximately four million people each year. A meta-analysis of 9,559 individuals found that the effects of *H. pylori* on type 1 and 2 diabetes and diabetes mellitus (both types) were 1.19 (95% CI = 0.98–1.45), 1.43 (95% CI = 1.11–1.85) and 1.17 (95% CI = 0.94–1.45), respectively, indicating that *H. pylori*-infected individuals would have a higher risk of diabetes. According to an analysis of geographical subpopulation regions, the infection risk of *H. pylori* in the Asian population was slightly higher than that in other populations (Mansori et al., 2020). In contrast, a cross-sectional study showed that there was no significant correlation between *H. pylori* and diabetes, though it has been estimated that *H. pylori* may be associated with an increased risk of diabetes in Chinese females (Man et al., 2020). Moreover, an ongoing clinical study may help reveal the association between diabetes and *H. pylori* infection by investigating the genome-wide association of *H. pylori* infection (PLCO-989).

Obesity has become a crucial public health problem. The impact of *H. pylori* on obesity or overweight is still unclear. A meta-analysis including 22 articles and 178,033 samples showed that obesity was associated with *H. pylori*, which may increase the risk of obesity (OR = 1.2) (Xu et al., 2019). However, from a retrospective study of 3,039 subjects, *H. pylori* was not related to obesity or overweight observed in a Chinese population (*P* = 0.321) (Xu et al., 2017). More investigation of the relationship between *H. pylori* infection and obesity are still needed.

The relation of *H. pylori* with autoimmune thyroid diseases (AITDs) also needs more research to clarify. A meta-analysis of 15 articles that included 3,046 cases showed that *H. pylori* was positively correlated with HT and GD (HT: 95% CI: 1.44–3.23, OR = 2.16; GD: 95% CI: 1.68–4.61, OR = 2.78), and CagA+ *H. pylori* was positively related to AITD (95% CI: 1.07–3.70, OR = 1.99) (Hou et al., 2017). Nevertheless, another study proposed that this pathogenesis might be caused by molecular mimics and an increased inflammatory state (Figura et al., 2019; Supplementary Table S3).

## Nerve disease

Alzheimer’s disease (AD), as a kind of nerve disease characterized by neurodegeneration, has also been studied for a possible association with *H. pylori* infection. Beydoun et al. (2018) found a direct relationship between AD mortality and *H. pylori* seropositivity in their retrospective cohort study that included 16,970 participants. In addition, a systematic study also revealed that AD may be associated with gastrointestinal microbiota dominated by *H. pylori* (Katsinelos et al., 2019).

Parkinson’s disease (PD) is the second most common neurodegenerative disorder in the world. Although the pathogenesis of PD remains unclear, *H. pylori* eradication was found to intensively improve the clinical symptoms of PD in a prospective cohort study. *H. pylori* eradication not only increased the normal motor function time (also known as ‘on’ time) of the day, but also improved gastrointestinal symptoms and reduced fatigue symptoms (Lolekha et al., 2021). Another case–control study found that the positive serum of *H. pylori* was related to the adverse reaction and higher dosage of levodopa, and *H. pylori* eradication improved the prognosis of patients (Mridula et al., 2017). A meta-analysis of 13 studies also found that *H. pylori* infection was significantly associated with adverse drug response, higher levodopa equivalent daily dose (LEDD) and severer motor symptoms in PD patients (Zhong et al., 2022).

A descriptive analytical cross-sectional study in Iran showed that *H. pylori* was related to the etiology of restless legs syndrome (RLS). Proinflammatory cytokines released by *H. pylori* infection, such as IL-6, have been shown to increase production of hepcidin, which affects iron transport in healthy human, resulting in an iron deficiency in the CNS and causing RLS (Rezvani et al., 2018).

The etiology of multiple sclerosis (MS) is the complex interaction of environmental and genetic factors. Bacterial exposure has been identified as one of the many pathogenic factors of MS (Cossu et al., 2018). As shown in a meta-analysis conducted in Western countries, the presence of bacteria was negatively correlated with MS (Jaruvongvanich et al., 2016). In Asian countries, *H. pylori* antigen antibodies were more common in patients with aquaporin 4 antibody-positive neuromyelitis optica spectrum disorders (NMOSDs) but negative in patients with MS (Yoshimura et al., 2013). The above results suggested that *H. pylori* may be a protective factor by manipulating pattern-recognition receptors (PRRs) (Efthymiou et al., 2017) and inhibiting Th1/Th17-cell responses (Salama et al., 2013). A recent seroprevalence study showed that antibodies against VacA were frequently detected in patients with secondary progressive MS (Efthymiou et al., 2017). Aside from the local role of *H. pylori*, the direct regulation was observed in the brain-intestinal axis (Kountouras et al., 2015).

Guillain–Barré syndrome (GBS) is a serious peripheral nerve autoimmune demyelinating disease that often occurs after bacterial infection. A meta-analysis revealed that there was an intensive relationship between GBS and *H. pylori* antibodies, especially in cerebrospinal fluid, suggesting that *H. pylori* is significant in GBS pathophysiology (Dardiotis et al., 2020; Supplementary Table S4).

## Ophthalmic disease

Glaucoma is a leading cause of blindness worldwide. A meta-analysis that included 15 studies and 2,664 participants found that *H. pylori* infection was associated with non-heterogeneous glaucoma (Doulberis et al., 2020b). Following *H. pylori* eradication therapy, a significant (*p* = 0.005) reduction in intraocular pressure (IOP) was found after 2 months of follow-up, showing that *H. pylori* eradication may be positive in glaucoma therapy (Ala et al., 2020).

A meta-analysis found a higher *H. pylori* prevalence among central serous chorioretinopathy (CSR) patients (Bagheri et al., 2017). In addition, some studies have indicated that CagA antigen antibodies might cross-react with vascular endothelial antigens to promote the occurrence of vascular wall injury and atherosclerosis (Franceschi et al., 2002). As atherosclerosis is one of the most significant risk factors for CSR, *H. pylori* may play a pathogenic role in CSR and injure the vascular endothelium through similar antigens and cross-reactivity (Supplementary Table S5).

## Dermatological disease

Alopecia areata is an inflammatory alopecia mediated by immunity that appears in all age and ethnic groups. The results of a case–control study including 162 examples showed that *H. pylori* infection may have a pathogenic effect on alopecia areata (Behrangi et al., 2017). *H. pylori* can promote chronic immune responses and local inflammatory, leading to sustained release of inflammatory mediators including PAF, LTC4, IFN-γ, TNF-α, and IL-1. These mediators may contribute to the occurrence of alopecia areata.

Besides, a meta-analysis of 11 studies and 1,741 examples revealed that *H. pylori* was also associated with psoriasis and that *H. pylori*+ individuals had a higher score on the Psoriasis Area and Severity Index (PASI) (Yu M. et al., 2019). However, a population-based longitudinal cohort study found no correlation between *H. pylori* and psoriasis (Wu et al., 2020). Thus, more studies are necessary to determine the relationship between psoriasis and *H. pylori*.

Similarly, a meta-analysis of 27 studies confirmed that *H. pylori* was related to the rosacea process (Yang, 2018), and *H. pylori*-infected individuals had a higher risk of suffering from rosacea.

Urticaria, a prevalent dermatological disease has also been reported to have a relation with *H. pylori*. Some studies found that the level of *H. pylori* antigens in individuals with chronic urticaria was significantly higher than that in controls. The eradication of *H. pylori* alleviated the symptoms of these patients, which supported an impact of *H. pylori* on pathogenesis (Erdem et al., 2020; Supplementary Table S5).

## Urinary disease

*Helicobacter pylori* is significantly related to immunoglobulin A (IgA) nephropathy, membranous nephropathy, Henoch–Schonlein purpura nephritis, diabetic nephropathy and other urinary diseases (Moriyama et al., 2007). *H. pylori* antigens were found in pathological tissues of these diseases (Li et al., 2013). A study indicated that *H. pylori* was probably a risk factor for kidney damage in patients with *H. pylori*+ peptic ulcers, and eradication of *H. pylori* may alleviate kidney damage and prevent chronic processes (Pan et al., 2019). Another study revealed that *H. pylori* infection may lead to a strong mucosal immune response and play a pathogenic role in IgA nephropathy based on renal tubular injury (Zhu et al., 2016; Supplementary Table S5).

## Reproductive disease

Previous studies have revealed that in men with fertility problems, the prevalence of *H. pylori* was much higher. Some immunocytochemical studies emphasized that serum samples from infected men (as well as anti-*H. pylori* hyperimmune serum) reacted with the equatorial segment and the flagella (especially abundant in tubulin) of sperm (Figura et al., 2002). However, in 2020, a cross-sectional study found that there was no difference in anti-Müllerian hormone (AMH) levels and sperm parameters in Chinese patients based on *H. pylori* infection history (Feng et al., 2020).

A cohort study showed that there was no significant relationship between subsequent prostate cancer risk and *H. pylori*-infected peptic ulcers (Fang et al., 2020). To date, the relationship of prostate cancer (PCa), benign prostatic hyperplasia (BPH), and *H. pylori* needs to be further studied.

Hyperemesis gravidarum (HG) is characterized by excessive vomiting and severe nausea that begins before the end of 22 weeks of pregnancy (World Health Organization, 2016). A study showed that in the stomach of women with HG, *H. pylori* was more prevalent, and there was a significant positive correlation between *H. pylori* serum levels and HG symptoms (Bustos et al., 2017; Supplementary Table S5).

## Other diseases

Laryngeal cancer is a serious disease threatening human health. A prospective controlled study found that in cases of *H. pylori* ureA gene-positive laryngeal cancer, 46.7–49.3% of 75 were also CagA positive. The CagA gene in laryngeal cancer greatly reduced the survival rate and increased the possibility of recurrence (Burduk, 2013).

Some studies revealed an association between oral diseases and *H. pylori* infection. Okuda et al. (2000) found the expression of *H. pylori* in the dental plaques in 12 of 54 *H. pylori* infected subjects. Moreover, a study reported that some oral samples expressed the *H. pylori* ureA gene, and the primary host of oral infection was identified as dental pulp (Iwai et al., 2019). The presence of *H. pylori* may be harmful to the oral environment. Recurrent aphthous stomatitis (RAS) is regarded as a recurrent painful ulcerative disease that regularly impacts mucosa in the oral cavity. Gao et al. (2021) reported a RAS case with a history of 24 years that was cured after treatment for *H. pylori*, indicating that eradication of *H. pylori* might relieve RAS symptoms and is a promising RAS therapy.

In addition to the standard drug regimen, the clinical practice of appending antidepressants to the treatment of *H. pylori* eradication is not quite explicit. A meta-analysis that included three RCTs, two review articles, one cohort study, four prospective studies, and eight cross-sectional studies found that individuals with functional dyspepsia who did not improve after *H. pylori* eradication (Al Quraan et al., 2019). Another study found that stress/anxiety/depression (SAD) and *H. pylori* infection were significantly prevalent in patients with functional dysplasia (FD) (Kabeer et al., 2017). A cohort study showed that in the general Chinese adult population, *H. pylori* infection was related to depressive symptoms in women but not men (Gu et al., 2019; Supplementary Table S5).

## Discussion

**FIGURE 2:**
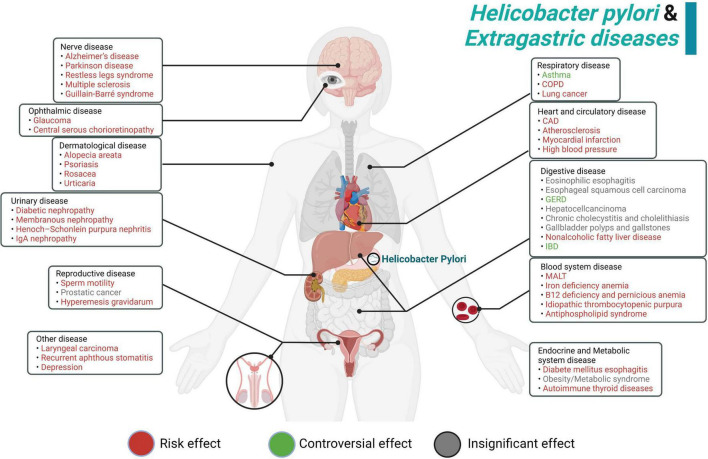
Summary schematic of the systematic effect of *H. pylori* infection. In red, the manifestations for which *H. pylori* infection represents a risk effect. In green, although some studies revealed that *H. pylori* showed a positive effect on these diseases, there are still many controversial aspects and further research is needed. In gray, we show the manifestations for which *H. pylori* infection shows an insignificant effect.

**FIGURE 3:**
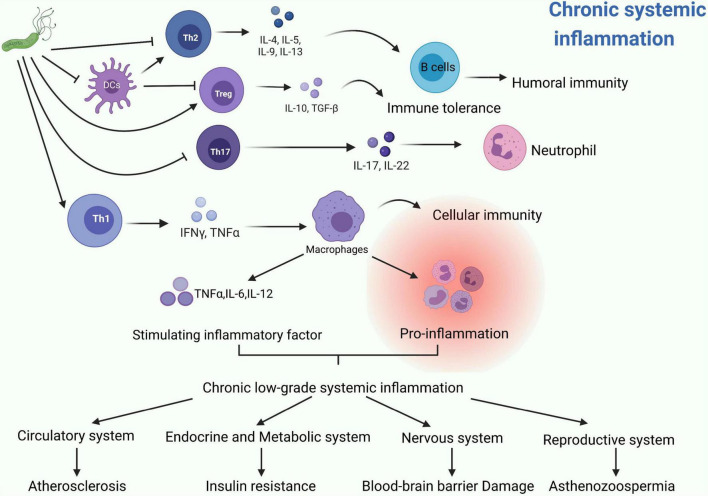
The possible common mechanisms by which *H. pylori* induces these systemic diseases can be summarized into two promising hypotheses: Hypotheses 1. Since *Helicobacter pylori* can induce several inflammatory factors, such as IL-1/2/6/8/10, TNF-α and IFN-γ, these factors may lead to chronic low-level systemic inflammation in the human body and ultimately represent diseases (Franceschi et al., 2014). Typical disorders due to *H. pylori*-induced inflammatory factors turbulence include: atherosclerosis (de Boer et al., 2000), insulin resistance (Cheng et al., 2017), blood-brain barrier damage, brain neurodegenerative disease (Efthymiou et al., 2017), and decreased sperm motility (Figura et al., 2002).

**FIGURE 4:**
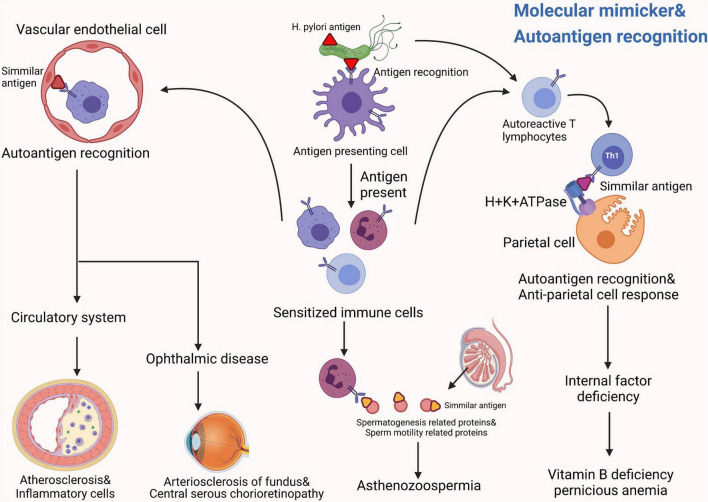
The possible common mechanisms by which *H. pylori* induces these systemic diseases can be summarized into two promising hypotheses: Hypotheses 2. *H. pylori* antigen, like the antigen components of host leads to molecular mimicker and cross-antigen reactions, which cause autoimmune attacks and relevant diseases (Chmiela and Gonciarz, 2017). Typical diseases that chiefly rely on this mechanism include: a cross reaction between the CagA antibody and the vascular wall induces atherosclerosis (Guo et al., 2007); *H. pylori* and gastric H+K+ATPase cross antigen contributes to vitamin B12 deficiency (Claeys et al., 1998); arteriosclerosis of fundus for autoimmune reaction induces central serous choroidal retinopathy (CSR) (Franceschi et al., 2002); cross-antigen reactivity between spermatogenesis-related proteins, sperm motility related proteins and *H. pylori* contributes to hypomotility of sperm (Figura et al., 2002).

Previously, *H. pylori* infection was mostly considered as a risk factor for gastric disorders. However, growing evidences show that *H. pylori* infection presents more complexity and tends to be associated with almost every system in the human body. From our perspective, *H. pylori* can produce many kinds of bacterial toxins and induce numerous extragastric diseases in the human body, such as asthma, COPD, ITP and psoriasis. We summarized these diseases in terms of the human system, listed them methodically in this article and showed a schematic diagram (Figure 2). Interestingly, recent studies mentioned in this review partially elucidate the potential pathogenesis of these extragastric diseases caused by *H. pylori* infection. We synthesized the results of these studies and proposed two promising hypotheses. (i) Since *H. pylori* can induce several inflammatory factors, such as IL-1/2/6/8/10, TNF-α and IFN-γ, these factors may lead to chronic low-level systemic inflammation in the human body and ultimately represent diseases. Typical disorders due to *H. pylori*-induced inflammatory factor turbulence include atherosclerosis, insulin resistance, blood-brain barrier damage, brain neurodegenerative disease and decreased sperm motility (Figure 3). (ii) *H. pylori* antigen, like the antigen components of host leads to molecular mimicker and cross-antigen reactions, which cause autoimmune attacks and relevant diseases. Typical diseases chiefly rely on this mechanism, including cross reaction between CagA antibody and vascular wall inducing atherosclerosis; *H. pylori* and gastric H+K+ATPase cross antigen contributes to vitamin B12 deficiency; arteriosclerosis of fundus for autoimmune reaction induces central serous choroidal retinopathy (CSR); and cross-antigen reactivity between spermatogenesis related proteins, sperm motility related proteins and *H. pylori* contributes to hypomotility of sperm (Figure 4). In a word, it is believed that the two hypotheses contribute to deciphering the reasons why *H. pylori* is associated with disorders in many systems of the human body (Franceschi et al., 2014; Chmiela and Gonciarz, 2017).

There are still some limitations of current studies that need to be improved. First, at present, the sample size of *H. pylori*-related extragastric diseases in most studies is generally insufficient. Larger sample sizes and broader clinical trials are beneficial to decipher the correlation between various clinical diseases and *H. pylori*, and the control of confounding factors is necessary. In addition, the pathogenic effect of *H. pylori* in some extragastric diseases, such as gastroesophageal reflux disease, asthma, and IBD, are still controversial (Figure 2). Some studies even proposed that *H. pylori* may have a certain protective effect on some diseases (such as GERD) (Scida et al., 2018). And most studies are only correlation studies without explanation of causality. The proof of Evidence-based medicine is not strong enough. It also needs to clarify causality with the help of animal model research of disease and in-depth molecular mechanism research. What’s more, the hypothesis (i) about systemic inflammation is limited for the heterogeneity of participants and the control of confounding factors is often incomplete (Kim T. J. et al., 2018). Therefore, the establishment of *H. pylori* infection model based on specific inflammatory markers (such as CRP and PLR) and the study of inflammatory-activated pathways are of great significance to reveal the systemic effects of *H. pylori*. Furthermore, there is high heterogeneity in the research on the relationship between *H. pylori* and the development of autoimmune diseases, and the differences of their distribution patterns make the research results controversial. At present, it is found that *H. pylori* infection may increase susceptibility to autoimmune diseases by stimulating cell damage, chronic inflammatory, and polyclonal lymphocyte activation (Youssefi et al., 2021). Aside from that, several intervention variables, including antibiotic treatment, microbiota, and host genome polymorphism may also be involved in the self-recognition of anti-*H. pylori* antibodies. The pathogenesis of gastric immunity induced by *H. pylori*, including hypergastrinemia and hypochlorhydria, may lead to changes in gastrointestinal microbiome. Nevertheless, the exact potential mechanism needs to be further clarified to confirm the systematic effects of *H. pylori* infection. In addition, in current clinical practice, the first-line treatment for most *H. pylori*-related extragastric diseases remains *H. pylori* eradication. However, *H. pylori* treatment to prevent allergic asthma and coronary artery disease has showed promising clinical outcomes (Zuin et al., 2016; Zhou et al., 2017). Thus, it is worth exploring that *H. pylori* preventive control strategies may be valuable for the contribution of other extragastric diseases. In general, most of previous articles on the extragastric diseases caused by *H. pylori* infection have limitations on the finite sample size, unclear pathogenic mechanism, and the limitation of *H. pylori* detection means (Supplementary Table S6).

*Helicobacter pylori* infection can induce several extragastric diseases through many pathways, and different types of *H. pylori* may contribute to different kinds of diseases because of their specific bacterial toxins and pathogenies. It is generally accepted that the systemic effects of *H. pylori* infection should not be neglected. Although *H. pylori* has been discovered over more than 100 years ago, many aspects of *H. pylori* still need further studies. For clinical practitioners, the impact of *H. pylori* infection on extragastric diseases should be taken into more consideration.
