# Supplementary material for: Helicobacter pylori and unignorable extragastric diseases: Mechanism and implications
Source: Front Microbiol. 2022 Aug 4;13:972777. doi: 10.3389/fmicb.2022.972777 (PMC9386483; doi:10.3389/fmicb.2022.972777)
Supplement: Supplementary file 1 [file Data_Sheet_1.docx]

| Table S1 Conflicting reports on the involvement of *H. pylori* in respiratory and circulatory diseases | | | | | | | | | | | | | | | | | | | | | | |
| --- | --- | --- | --- | --- | --- | --- | --- | --- | --- | --- | --- | --- | --- | --- | --- | --- | --- | --- | --- | --- | --- | --- |
| **Respiratory disease** | **Correlated** | | | **Study type** | | | | | | **p/OR-values** | | | | | **Noncorrelated** | | | **Study type** | | | **p/OR-values** | |
| Asthma | Zuo et al | | | | review | | | | |  | | | Miftahussurur et al | | | | | review | |  | | |
|  | Wang et al | | | | cohort study | | | p<0.001 | | | | |  | | | | |  | |  | | |
| COPD | Sze et al | | | | cohort study | | | | | p<0.001 | | | Lee et al | | | | cohort study | | | | | p=0.549 |
| Lung cancer | Xu et al | | | | observational retrospective study | | | | p<0.05 | | | |  | | | | |  | |  | | |
|  | GonzAlez et al | | | | | | review | | |  | | |  | | | | |  | |  | | |
| **Circulatory disease** | **Correlated** | | | | **Study type** | | | | | **p/OR-values** | | | **Noncorrelated** | | | | | **Study type** | | | **p/OR-values** | |
| Coronary artery disease | Xia et al | | multicenter study | | | | | | | | | p<0.005 | |  | | | |  | | |  | |
|  | Sharma et al | | | | review | | | | |  | | | | |  |  | | |  | | | |
|  | Byrne et al | | | | review | | | | |  | | | | |  |  | | |  | | | |
|  | de Boer et al | | | | review | | | | |  | | | | |  |  | | |  | | | |
| Myocardial infarction | | Liu et al | | | meta-analysis | | | | | | | OR=2.80 | | |  |  | | |  | | | |
| Carotid atherosclerosis | | Zhang et al | | | cohort study | | | | | | | p=0.028 | | |  |  | | |  | | | |
|  |  | Yu et al | | | | cohort study | | | | | p=0.0052 | | | |  |  | | |  | | | |
| High blood pressure | | Wan et al | | | cohort study | | | | | | | OR=1.23 | | |  |  | | |  | | | |

| Table S2 Conflicting reports on the involvement of *H. pylori* in digestive diseases | | | | | | | | | | | | | | | | | |
| --- | --- | --- | --- | --- | --- | --- | --- | --- | --- | --- | --- | --- | --- | --- | --- | --- | --- |
| **Digestive disease** | **Correlated** | | | **Study type** | | | | **p/OR-values** | | | | **Noncorrelated** | | **Study type** | **p/OR-values** | | |
| Eosinophilic esophagitis | | |  | |  | | | |  | | | Doulberis et al | | review | | |  |
|  |  | | | |  | | | |  | Molina-Infante et al | | | | multicenter study | | | p>0.05 |
| Esophageal squamous cell carcinoma | | Poyrazoglu et al | | | retrospective study | | | | p<0.0001 | | | Gao et al | | review | | |  |
| Gastroesophageal reflux disease | Jie et al | | | | retrospective study | | | | p<0.05 | | |  | |  | | |  |
|  | Liu et al | | | | prospective clinical study | | | | p<0.05 | | |  | |  | | |  |
| Hepatocellular  carcinoma |  | | | |  | | | |  | | | Okushin et al | | review | | |  |
| Chronic cholecystitis and cholelithiasis | Wang et al | | | | review | | | |  | | |  |  | | |  | |
| Cholecystic polypus and gallstones | Xu et al | | | | retrospective study | | | | p<0.05 | | Zhang et al | | | retrospective study | | | p>0.05 |
| Nonalcoholic fatty liver disease | Liu et al | | | | meta-analysis | | | | p<0.001 | | Fan et al | | | cross sectional study | | | OR=0.9 |
|  | Cheng et al | | | | | review | | |  | |  | | |  | | |  |
| Inflammatory bowel disease | Imawana et al | | | | | | meta-analysis | | p<0.001 | |  | | |  | | |  |
|  | Tepler et al | | | | | | meta-analysis | | p<0.05 | |  | | |  | | |  |
| Chronic hepatitis B disease | Wang et al | | | | | | meta-analysis | | p<0.001 | |  | | |  | | |  |

| Table S3 Conflicting reports on the involvement of *H. pylori* in the blood system and endocrine diseases | | | | | | | | | | | | | | | |
| --- | --- | --- | --- | --- | --- | --- | --- | --- | --- | --- | --- | --- | --- | --- | --- |
| **Blood system disease** | **Correlated** | | | **Study type** | | **p/OR-values** | | | **Noncorrelated** | | | **Study type** | **p/OR-values** | | |
| Mucosa-associated lymphoid tissue | Zhang et al | | | review | |  | | |  | | |  |  | | |
|  | Kuo et al | | | cohort study | | p<0.05 | | |  | | |  |  | | |
|  | Malfertheiner et al | | | consensus report | |  | | |  | | |  |  | | |
| Iron deficiency anemia | Flores et al | | cell experiment | | | | p<0.05 | |  | | |  |  | | |
| B12 deficiency and pernicious anemia | Cohen et al | | | | prospective clinical study | | p<0.05 | |  | | |  |  | | |
|  | Claeys et al | | | cohort study | | | p<0.01 | |  | | |  |  | | |
| Idiopathic thrombocytopenic purpura | Kim et al | | | meta-analysis | | p<0.05 | | | | |  |  |  | | |
|  | Lei et al | | | animal experiment | | p<0.05 | | | | |  |  |  | | |
| Antiphospholipid syndrome | Cicconi et al | case report | | | | | |  | |  | |  |  | | |
| **Endocrine disease** | **Correlated** | | | **Study type** | | **p/OR-values** | | | **Noncorrelated** | | | **Study type** | **p/OR-values** | | |
| Diabetes | Mansori et al | | | meta-analysis | | OR=1.27 | | |  | | |  |  | | |
|  | Man et al | | | cross sectional study | | p=0.05 | | |  | | |  |  | | |
| Obesity | Xu et al | | | meta-analysis | | OR=1.20 | | | Xu et al | | | retrospective study | | p=0.729 | |
| Autoimmune thyroid diseases | Hou et al | | | meta-analysis | | OR=2.25 | | |  | | |  | | |  |
|  | Figura et al | | | cohort study | | p<0.001 | | |  | | |  |  | | |

| Table S4 Conflicting reports on the involvement of *Helicobacter pylori* in nerve diseases | | | | | | | | | | | | | | |
| --- | --- | --- | --- | --- | --- | --- | --- | --- | --- | --- | --- | --- | --- | --- |
| **Nerve disease** | **Correlated** | **Study type** | | **p/OR-values** | | | | **Noncorrelated** | | | | | **Study type** | **p/OR-values** |
| Alzheimer’s disease | Beydoun et al | cohort study | | p<0.05 | | | |  | | | | |  |  |
|  | Katsinelos et al | review |  | | |  | | |  | | |  | | |
| Parkinson’s disease | Lolekha et al | cohort study | | | p<0.05 | |  | |  |  | | | | |
|  | Mridula et al | cohort study | | | p<0.0001 | |  | |  | |  | | | |
|  | Zhong et al | meta-analysis | | | p<0.01 | |  | |  | |  | | | |
| Restless legs syndrome | Rezvani et al | cross sectional study | | | p<0.05 | |  | |  | |  | | | |
| Multiple sclerosis | Yoshimura et al | cohort study | | | p<0.05 | |  | |  | |  | | | |
|  | Efthymiou et al | cohort study | | | p<0.05 | |  | |  | |  | | | |
|  | Kountouras et al | review | | |  | |  | |  | |  | | | |
| Guillain–Barré syndrome | Dardiotis et al | meta-analysis | | | OR=2.31 | |  | |  | |  | | | |

| Table S5 Conflicting reports on the involvement of *H. pylori* in ophthalmic/dermatological/urinary/reproductive/other diseases | | | | | | | | | | | | | | | | | | | | | | |
| --- | --- | --- | --- | --- | --- | --- | --- | --- | --- | --- | --- | --- | --- | --- | --- | --- | --- | --- | --- | --- | --- | --- |
| **Ophthalmic disease** | | | | **Correlated** | | | | **Study type** | | | **p/OR-values** | | | | **Noncorrelated** | | | | **Study type** | **p/OR-values** | | |
| Glaucoma | Doulberis et al | | | | | | | | review | | |  | | |  | |  | | |  | | |
|  | | | | Ala et al | | | | cohort study | | | | p<0.01 | | |  | |  | | |  | | |
| Central serous chorioretinopathy | | | | Bagheri et al | | meta-analysis | | | | | | p<0.01 | | |  | | |  | | |  | |
| **Dermatological disease** | | | | **Correlated** | | | **Study type** | | | | | **p/OR-values** | | | **Noncorrelated** | | | | **Study type** | | **p/OR-values** | |
| Alopecia areata | Behrangi et al | | | | | | | | cohort study | | | | p=0.011 | | |  | | |  | | |  |
| Psoriasis | | | | | Yu et al | | | meta-analysis | | | | | OR=1.19 | | | Wu et al | | | cohort study | | | p=0.3227 |
| **Urinary disease** | | | | | **Correlated** | | | **Study type** | | | | | **p/OR-values** | | | **Noncorrelated** | | | **Study type** | | | **p/OR-values** |
| Urticaria | | Erdem et al | | | | | | | cohort study | | | | | p<0.05 | |  | | |  | | |  |
| Membranous nephropathy | Moriyama et al | | | | | | | | cross sectional study | | | | | p<0.05 | |  | | |  | | |  |
| Glomerulonephritis | | | | | Li et al | | | cohort study | | | | | p<0.001 | | |  | | |  | | |  |
| Kidney damage | | | | | Pan et al | | | cohort study | | | | | p<0.01 | | |  | | |  | | |  |
| IgA nephropathy | | | Zhu et al | | | | | cohort study | | | | | p<0.05 | | |  | | |  | | |  |
| **Reproductive disease** | | | | | **Correlated** | | | **Study type** | | | | | **p/OR-values** | | | **Noncorrelated** | | | **Study type** | | | **p/OR-values** |
| Low sperm motility | | | Figura et al | | | | | cohort study | | | | | p<0.001 | | | Feng et al | | | cross sectional study | | | p>0.05 |
| Prostatic cancer | | | | |  | | |  | | | | |  | | | Fang et al | | | cohort study | | | p<0.24 |
| Hyperemesis gravidarum | | | | | Bustos et al | | | | | review | | |  | | |  | | |  | | |  |
| **Other disease** | | | | | **Correlated** | | | **Study type** | | | | | **p/OR-values** | | | **Noncorrelated** | | | **Study type** | | | **p/OR-values** |
| Larynx cancer | | | | | Burduk et al | | | prospective study | | | | | p<0.05 | | |  | | |  | | |  |
| Oral infection | | | | | Okuda et al | | | cross sectional study | | | | | p<0.05 | | |  | | |  | | |  |
|  | | | Iwai et al | | | | | cohort study | | | | | p<0.01 | | |  | | |  | | |  |
| Recurrent aphthous stomatitis | | | | | Gao et al | | | case reports | | | | |  | | |  | | |  | | |  |
| Depression | | | | | Al et al | | | meta-analysis | | | | | p<0.05 | | |  | | |  | | |  |
|  | | | | | Gu et al | | | cross sectional study | | | | | p<0.05 | | |  | | |  | | |  |
